# Artificially Cultivated *Ophiocordyceps sinensis* Alleviates Diabetic Nephropathy and Its Podocyte Injury via Inhibiting P2X7R Expression and NLRP3 Inflammasome Activation

**DOI:** 10.1155/2018/1390418

**Published:** 2018-11-11

**Authors:** Chao Wang, Xiao-xia Hou, Hong-liang Rui, Li-jing Li, Jing Zhao, Min Yang, Li-jun Sun, Hong-rui Dong, Hong Cheng, Yi-Pu Chen

**Affiliations:** Division of Nephrology, Beijing Anzhen Hospital, Capital Medical University, Beijing 100029, China

## Abstract

**Background/Aims:**

It is known that chronic low-grade inflammation contributes to the initiation and development of both diabetes and diabetic nephropathy (DN), so we designed this study to investigate the role of P2X7R and NLRP3 inflammasome in DN pathogenesis and the antagonistic effects of artificially cultivated *Ophiocordyceps sinensis* (ACOS).

**Methods:**

A rat model of DN caused by high-fat-diet feeding and low-dose streptozotocin injection and a mouse podocyte injury model induced by high-glucose (HG) stimulation were established, and the intervention effects of ACOS on them were observed. The biological parameters of serum and urine and the pathological manifestations of kidney tissue were examined. The expression of mRNA and protein of P2X7R and NLRP3 inflammasome (NLRP3, ASC, and caspase-1) and downstream effectors (IL-1*β* and IL-18), as well as podocyte-associated molecules, was determined by real-time quantitative PCR and Western blot assay, respectively.

**Results:**

The DN rats showed to have developed insulin resistance, elevated fasting blood glucose, increased urinary protein excretion, and serum creatinine level as well as corresponding glomerular pathological alterations including podocyte damages. ACOS significantly antagonized the above changes. The experiments *in vivo* and *in vitro* both displayed that the mRNA and protein expression of P2X7R, NLRP3, ASC, caspase1 (procaspase-1 mRNA in the gene level and active caspase-1 subunit P10 in the protein level), IL-1*β*, and IL-18 was significantly upregulated and the mRNA and protein expression of podocyte-associated molecules was significantly changed (downregulation of nephrin, podocin, and WT-1 expression and upregulation of desmin expression) indicating podocyte injury in the kidney tissue of DN rats and in the HG-stressed mouse podocytes, respectively. ACOS also significantly antagonized all the above changes.

**Conclusion:**

Our research work suggests that P2X7R and NLRP3 inflammasome are involved in the pathogenesis of DN, and ACOS can effectively inhibit the high expression of P2X7R and the activation of NLRP3 inflammasome, which may contribute to the therapeutic effects of *Ophiocordyceps sinensis*.

## 1. Introduction

The global prevalence of diabetes has dramatically increased over the recent decades. The International Diabetes Federation reported that the overall prevalence of diabetes was estimated to be 8.8% in people aged 20~79 years worldwide in 2015, which was predicted to rise to 10.4% in 2040 [1]. The China Noncommunicable Disease Surveillance Group reported that the overall prevalence of diabetes was estimated to be 11.6% and the prevalence of prediabetes in Chinese adult population aged 18 years or older was estimated to be 50.1% in 2010 [2]. Diabetic nephropathy (DN), also known as diabetic kidney disease (DKD), is one of the most common chronic microvascular complications of diabetes, which is the leading cause of end-stage renal disease (ESRD) in developed countries and the second cause of ESRD in China [3]. So, diabetes with its kidney complication, DN, has become a worldwide public health problem that seriously threatens human health.

DN is a glomerular disease. Now, it has been recognized that podocyte injury is a pivotal event in the pathogenesis of DN [4]. However, the underlying mechanism of podocyte injury in DN has not been fully clarified. A growing number of studies show that diabetes is characterized by chronic low-grade inflammation, referred to as “metaflammation,” which is a relevant factor contributing to the initiation and development of both diabetes and its complications including DN [5–7]. In the past years, it was discovered that the expression of purinergic 2X7 receptor (P2X7R) was significantly upregulated in glomerular cells, mainly in podocytes, in diabetic rat models [8]. It was also observed that the nucleotide-binding oligomerization domain-like receptor protein 3 (NLRP3) inflammasome activation in glomerular cells including podocytes was associated with the onset of DN, while NLRP3 inflammasome deficiency or inhibition could ameliorate DN in mouse models [7, 9]. The above studies have drawn our attention to the pathogenic role of P2X7R and NLRP3 inflammasome in DN.

P2X7R is a member of the P2XR family. It is characterized by the response to extracellular ATP (eATP), with a rapid opening of a ligand-gated cation channel, resulting in potassium ion (K^+^) efflux and intracellular potassium depletion. When the level of intracellular potassium reduces below the threshold of 90 mM, the assembly and activation of NLRP3 inflammasome are initiated [10–12]. The NLRP3 inflammasome is an intracellular multiprotein complex consisting of NLRP3, apoptosis-associated speck-like protein containing a caspase recruitment domain (ASC, adaptor protein), and cysteinyl aspartate specific protease-1 (caspase-1, effect protein). Caspase-1 usually exists as an inactive precursor procaspase-1, which can be self-enzymatically hydrolyzed to its active forms, P10 and P20 subunits, during the assembly and activation of NLRP3 inflammasome. The active P10/P20 subunits can turn the inactive precursors of interleukin 1 beta (IL-1*β*) and IL-18 into the mature forms to induce inflammation [13–15]. In this study, we investigated the relationship between P2X7R with its downstream NLRP3 inflammasome and DN and inspected the effects of *Ophiocordyceps sinensis* on them.

**Figure 1:**
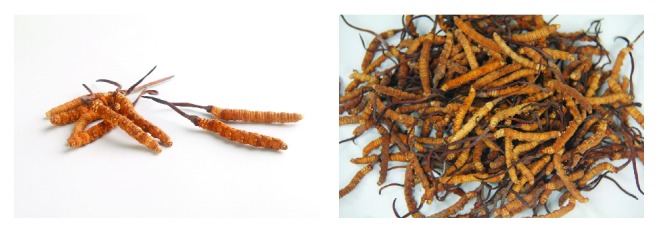
Artificially cultivated *Ophiocordyceps sinensis* (ACOS). *Ophiocordyceps sinensis* is a fungus-caterpillar complex formed after the fungus infects the larva of the moth that belongs to Hepialidae. The black part of the complex is the fungal part that is called the fruiting body and consists of stromatophore and stroma; the yellowish-brown part is the dead larva body that is filled with mycelia, called the sclerotium. The *Ophiocordyceps sinensis* in this photo is the ACOS, which has been produced through industrialized artificial cultivation in China now.

*Ophiocordyceps sinensis* (once called *Cordyceps sinensis*), a fungus-caterpillar complex formed after the fungus infects the larva of the moth that belongs to Hepialidae, is a well-known traditional Chinese medicine and has been used for centuries in China and Asian countries. Clinical practice and experimental studies have shown that it is effective in the treatment of many kinds of kidney diseases including DN [16–18]. However, so far, there have been no reports of the effects of *Ophiocordyceps sinensis* on P2X7R and NLRP3 inflammasome, as we did in this study. Wild *Ophiocordyceps sinensis* is now too scarce to meet the medical needs in China. So, artificially cultivated *Ophiocordyceps sinensis* (ACOS) has been highly expected for a long time. Fortunately, it has finally succeeded in recent years (Figure 1) [19–21]. In this study, we utilized the ACOS instead of wild *Ophiocordyceps sinensis* for the experiments *in vivo* and *in vitro*, which is the first experimental study of ACOS in the treatment of diseases.

In this study, we established a rat model of DN caused by type 2 DM and a mouse podocyte injury model induced by high-glucose (HG) stress and then studied the role of P2X7R and NLRP3 inflammasome in the pathogenesis of DN and the antagonistic effects of ACOS by using these models.

## 2. Materials and Methods

### 2.1. Animals and Grouping

Thirty-two male Sprague-Dawley rats weighing 180–200 g at the age of 6 weeks were purchased from Vital River Laboratory Animal Technology Co. (Beijing, China) and were housed in an animal room of specific-pathogen-free cleanliness grade with 50–60% humidity at temperature 20–26°C. Rats were randomly and equally divided into the following 4 groups: control group, DN model group, intervention group with a low dose of ACOS, and intervention group with a high dose of ACOS (HEC Pharm Co., China). The rats in the control group were fed with ordinary chow (energy ratio: fat—12.11%, protein—22.47%, and carbohydrates—65.42%), while the rats in the other three groups were fed with high-fat chow (energy ratio: fat—45.65%, protein—16.46%, and carbohydrates—37.89%). At the end of the 4th week, the insulin resistance index (IRI) was measured with the HOMA-IR formula in the rats fed with high-fat chow. After insulin resistance was confirmed, the rats in the DN model group and two intervention groups were intraperitoneally injected with streptozotocin (Sigma, USA) in a single dose of 35 mg/kg, while the rats in the control group were only injected with an equivalent volume of buffer. 72 h after the injection, the fasting blood glucose (FBG) of each rat was tested and rats are considered to have type 2 DM when their FBG level is >11.1 mmol/L. From the 5th week, the rats in the low- and high-dose intervention groups were given ACOS by gavage in a dose of 2.5 g/kg (LD-ACOS group) and 5.0 g/kg (HD-ACOS group), respectively, every day for 8 weeks, while the rats in the control and model groups were given the equal volume of tap water by gavage every day for 8 weeks.

### 2.2. Biological Parameters

Body weight was measured at baseline and at the 4th and 13th week. Kidney weight was measured after the rat was sacrificed, and then the ratio of kidney weight/body weight (KW/BW) of each rat was calculated. Urinary protein excretion of 24 h urine sample was tested at baseline and the 13th week. Serum creatinine (SCr) was detected at the 13th week. FBG was detected at the 4th and 13th week and also at 72 h after streptozotocin injection. Glycated hemoglobin (HbA1c) was measured at the 13th week. Fasting insulin was detected at the 4th and 13th week, and then IRI was calculated using the HOMA-IR formula: IRI = fasting blood glucose (mmol/L) × fasting insulin (mIU/L)/22.5 [22, 23].

### 2.3. Pathological Examination

After the renal tissue was conventionally processed, the 3 *μ*m thick sections were stained with a periodic acid-Schiff reagent for light microscopy. Twenty images of glomerular maximal profiles with a vascular pole and/or urinary pole were taken under a high-power microscope (×400, Olympus, Japan) and were analyzed by Nikon NIS-Elements BR image analysis software (Nikon, Japan). The length (*μ*m) of the two longest perpendicular diameters in every glomerular capillary tuft without Bowman's space was measured, and then the mean value was calculated. The areas of the glomerular mesangial region and capillary tuft were also measured, and then the relative area of the mesangial region (%) was calculated according to the formula: area of the mesangial region/area of the capillary tuft × 100%.

After the renal tissue was processed according to standard techniques. The ultrathin sections were stained with uranium acetate-lead citrate for electron microscopy. For each specimen, ten photographs (×20,000 magnification) covering different regions in the glomerular cross section were taken separately. The length (*μ*m) of the peripheral GBM was measured, and the number of slit pores overlying this GBM length was counted by Nikon NIS-Elements BR image analysis software (Nikon, Japan). The mean of the foot process width $\left({\bar{W}}_{FP}\right)$ was calculated as follows [24]:

(1) $$\begin{array}{c} {\bar{W}}_{FP}=\frac{\pi }{4}\cdot \frac{\sum GBM\;length}{\sum slits}, \end{array}$$

where ∑slits is the total number of slits counted and ∑GBM length is the total GBM length measured in one glomerulus.

### 2.4. Double Immunofluorescence Staining

For the double staining of an indirect immunofluorescence assay of P2X7R/NLRP3 and podocyte marker synaptopodin, frozen renal tissues of rats were cut into 5 *μ*m thick sections. A rabbit anti-P2X7R polyclonal antibody (1 : 100 dilution, Alomone) or rabbit anti-NLRP3 polyclonal antibody (1 : 100 dilution, Novus) and mouse anti-synaptopodin monoclonal antibody (1 : 50 dilution, Santa Cruz) were used as primary antibodies. Rhodamine-labeled goat anti-rabbit immunoglobulins (Beijing Zhongshan) and FITC-labeled goat anti-mouse immunoglobulins (Beijing Zhongshan) were used as secondary antibodies, respectively. After staining, the tissue sections were observed with a fluorescent microscope (Nikon, Japan).

### 2.5. Podocyte Culture and Grouping

The conditionally immortalized mouse podocyte cell line was kindly provided by Professor Maria Pia Rastaldi (S. Carlo Hospital, University of Milan). Podocytes were incubated in RPMI-1640 medium (Thermo Fisher Scientific) containing 10% inactivated fetal bovine serum (FBS, Thermo Fisher Scientific) and 10 *μ*/mL interferon-*γ* (IFN-*γ*, Cell Signaling Technology) at 33°C in a humidified air with 5% CO_2_. When the cells reached 80–90% confluence, they were transferred to RPMI-1640 medium containing 10% inactivated FBS without IFN-*γ* and incubated at 37°C in a humidified air with 5% CO_2_ for 10–14 days to allow differentiation.

Well-differentiated podocytes were used for experiments. The podocytes were incubated in the RPMI-1640 medium with 5% inactivated FBS and grouped as follows: medium alone (control group), medium containing 30 mM glucose (Sigma) (HG group), medium containing 50 *μ*g/mL ACOS extract (HEC Pharm Co., China) (ACOS control group), and medium containing both 30 mM glucose and 50 *μ*g/mL ACOS extract (HG + ACOS group). According to the LDH release test, 30 mM glucose had no cytotoxic effects on cellular viability (Table 1S, see Supplementary Materials).

### 2.6. Reverse Transcription and Real-Time Quantitative PCR

Total RNA was extracted from rat renal cortex tissue or cultured podocytes using TRIzol reagent (Invitrogen) following the manufacturer's instructions. 2 *μ*g total RNA from each sample was reverse-transcribed to cDNA with EasyScript First-Strand cDNA Synthesis SuperMix (TransGen Biotech). The gene-specific primers (SBS Genetech) are listed in Tables 1 and 2. RT-PCR was performed using SYBR Green RT-PCR Master Mix (TransGen Biotech) according to the manufacturer's instruction. The GAPDH was set as the internal control gene in the animal and cellular experiments. The relative quantity of mRNA expression was calculated according to the formula: 2^−(targetgeneCt − GAPDHCt)^ × 10^3^, in which Ct was the threshold cycle number. All assays were repeated at least in triplicate independently.

### 2.7. Western Blot Assay

Total protein lysates were extracted from rat renal cortex tissue or cultured podocytes using RIPA lysis buffer (ComWin Biotech). Protein samples were sonicated five times for 1 s each, centrifuged at 12000 rpm for 10 min at 4°C, and then boiled for 5 min. Protein samples were separated by 10–12% sodium dodecyl sulphate-polyacrylamide gel electrophoresis (SDS-PAGE) and transferred to nitrocellulose membranes (General Electric Co.). After being blocked with 5% skim milk in phosphate-buffered saline with 0.1% Tween 20 for 1 h, the membranes were incubated with a primary antibody at 4°C overnight and then incubated with a secondary antibody at room temperature for 1 h. Details regarding primary and secondary antibodies are listed in Table 3. The blotted proteins were quantified using the Odyssey Infrared Imaging System (LI-COR Biosciences). *β*-Actin was set as an internal control. The relative expression level of each target protein was displayed as a ratio of target protein/*β*-actin protein. All the assays were performed at least in triplicate independently.

### 2.8. Statistical Analysis

All the data of continuous variables were represented as the mean ± SD and analyzed by using SPSS 21.0 statistical software. One-way ANOVA was used to test the differences among groups. Statistical significance was defined as *P* < 0.05.

## 3. Results

In the course of the experiment, each one rat died in the model group, LD-ACOS group, and HD-ACOS group. Therefore, at the end of the experiment, only the data of the remaining seven rats in the 3 groups were statistically analyzed.

### 3.1. Effects of ACOS on the Body Weight and the Ratio of KW/BW of the Rat DN Model

Body weight at baseline among the four animal groups had no statistical difference (247.4 ± 5.9 g, 254.6 ± 9.3 g, 277.8 ± 9.8 g, and 252.6 ± 8.4 g in the DN, LD-ACOS, HD-ACOS, and control groups, respectively, *P* > 0.05). At the 4th week, the body weight of rats in the three groups fed with high-fat chow (412.7 ± 30.7 g, 422.3 ± 27.3 g, and 409.7 ± 31.6 g in the DN, LD-ACOS, and HD-ACOS groups, respectively) was significantly higher than that in the control group (374.4 ± 25.0 g) (*P* < 0.05). At the 13th week, the body weight in the DN group and LD-ACOS group was significantly lower than that in the control group (*P* < 0.05), and the body weight in the HD-ACOS group was higher than that in the DN group, but the difference between them was not yet statistically significant (*P* > 0.05) (Table 4).

At the 13th week, the ratio of KW/BW in the DN group and two intervention groups was significantly higher than that in the control group (*P* < 0.05), while in the HD-ACOS group, the ratio was significantly lower than that in the DN group (*P* < 0.05) (Table 4).

### 3.2. Effects of ACOS on the Blood Glucose and Insulin Resistance of the Rat DN Model

At the 4th week, the FBG levels among the four groups had no statistical difference (6.11 ± 0.65 mmol/L, 6.03 ± 1.11 mmol/L, 5.91 ± 1.08 mmol/L, and 6.14 ± 0.79 mmol/L in the DN, LD-ACOS, HD-ACOS, and control groups, respectively, *P* > 0.05). The FBG levels of rats in the DN group and two intervention groups were all beyond 11.1 mmol/L at 72 h after streptozotocin injection. At the 13th week, the FBG levels in the DN group and two intervention groups were significantly higher than those in the control group (*P* < 0.01), while in the HD-ACOS group, the level was significantly lower than that in the DN group (*P* < 0.01) (Table 4).

In addition, at the 13th week, the HbA1c levels in the DN group and two intervention groups were significantly higher than those in the control group (*P* < 0.01), and the HbA1c level in the HD-ACOS group was lower than that in the DN group, but the difference between them was not yet statistically significant (*P* > 0.05) (Table 4).

At the 4th week, the levels of blood insulin and IRI in the three groups fed with high-fat chow were significantly higher than those in the control group (*P* < 0.05). At the 13th week, the blood insulin level in the DN group was significantly lower than that in the control group (*P* < 0.05) and the level in the HD-ACOS group was significantly higher than that in the model group (*P* < 0.05). The IRI levels in the DN group and two intervention groups were significantly higher than those in the control group (*P* < 0.01), while the level in the HD-ACOS group was significantly lower than that in the model group (*P* < 0.01) (Table 5).

### 3.3. Effects of ACOS on the Proteinuria and Renal Function of the Rat DN Model

Urinary protein excretion among the four groups was not statistically different at baseline (5.4 ± 1.6 mg/d, 4.3 ± 0.9 mg/d, 4.4 ± 1.0 mg/d, and 4.9 ± 1.2 mg/d in the DN, LD-ACOS, HD-ACOS, and control groups, respectively, *P* > 0.05). At the 13th week, the urinary protein excretion in the DN group and two intervention groups was significantly higher than that in the control group (*P* < 0.05), while the excretion in the two intervention groups was significantly lower than that in the DN model group (*P* < 0.05) (Table 4).

At the 13th week, the SCr levels in the DN group and two intervention groups were significantly higher than those in the control group (*P* < 0.05), while the level in the HD-ACOS group was significantly lower than that in the DN group (*P* < 0.05) (Table 4).

### 3.4. Effects of ACOS on the Renal Pathological Parameters of the Rat DN Model

**Figure 2:**
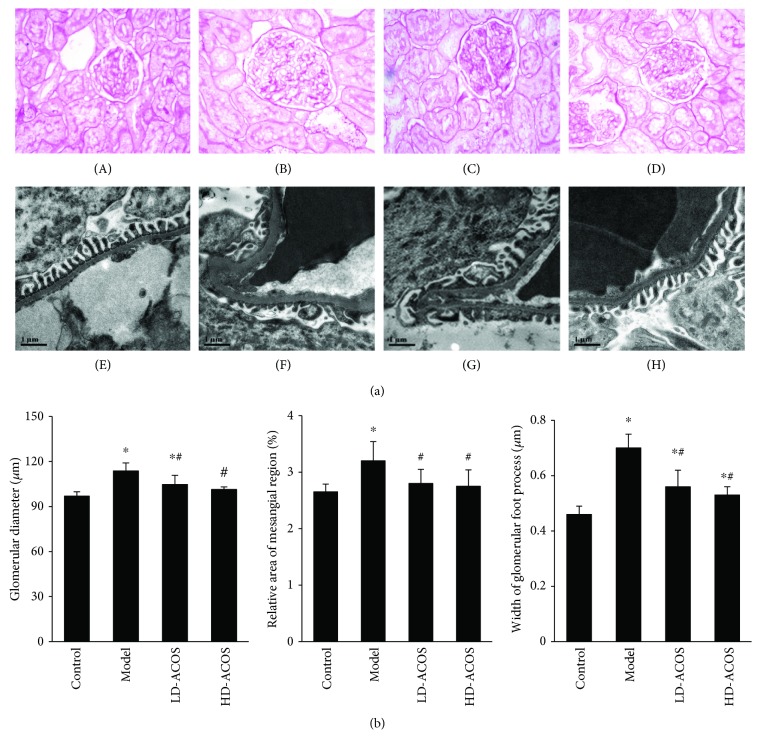
Effects of ACOS on the pathological parameters of the rat DN model. (a) Light microscopic images of glomerular size (PAS staining ×400) and electron microscopic images of foot processes of podocytes (×20,000). (A, E) Control group. (B, F) DN model group. (C, G) LD-ACOS group. (D, H) HD-ACOS group. (b) Histograms of the glomerular diameter, relative area of the mesangial region, and foot process width. Values are represented as mean ± SD. ^∗^ *P* < 0.05 vs. control group, ^#^ *P* < 0.05 vs. DN model group.

Light microscopy of kidney tissue showed that the average glomerular size, which was represented as an average glomerular diameter, in the DN group and LD-ACOS group was significantly larger than that in the control group (*P* < 0.05), while the size in the two intervention groups was significantly smaller than that in the DN group (*P* < 0.05). The relative area of the mesangial region in the DN group was significantly larger than that in the control group (*P* < 0.05), while the area in the two intervention groups was significantly smaller than that in the DN group (*P* < 0.05) (Figure 2).

Electron microscopy of kidney tissue indicated that the foot processes of podocytes appeared to experience segmental effacement and the average width of foot processes in the DN group and two intervention groups was significantly larger than that in the control group (*P* < 0.05), while the width in the two intervention groups was significantly smaller than that in the DN group (*P* < 0.05) (Figure 2).

### 3.5. Effects of ACOS on the Expression of Podocyte-Associated Molecules in the Renal Cortex of the Rat DN Model

**Figure 3:**
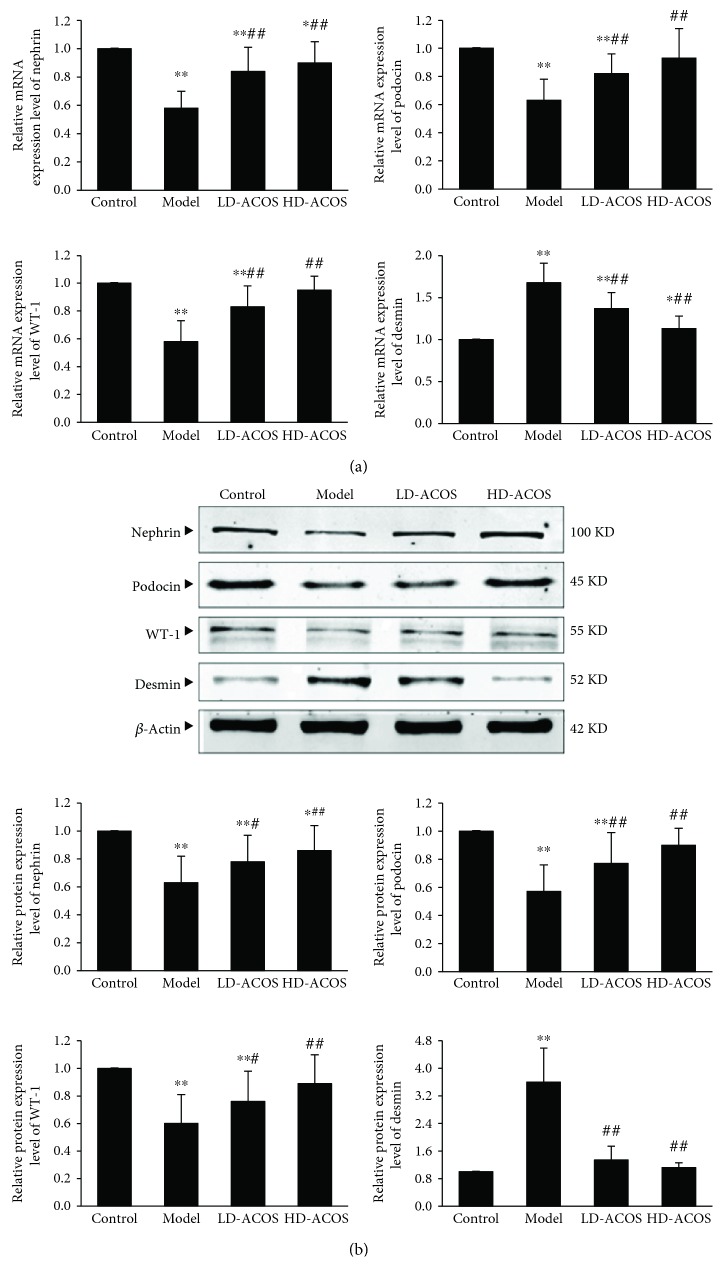
Effects of ACOS on the expression of podocyte-associated molecules in the rat DN model. (a) Total RNA was extracted from renal cortex tissue, and the relative mRNA expression levels of nephrin, podocin, WT-1, and desmin were measured by real-time quantitative PCR. (b) Renal cortex tissue was lysed, and total lysates were analyzed by the Western blot assay with antibodies against nephrin, podocin, WT-1, desmin, and *β*-actin, respectively. The relative protein expression level was expressed as the target protein/*β*-actin ratio. Values are represented as mean ± SD. ^∗^ *P* < 0.05 and ^∗∗^ *P* < 0.01 vs. control group, ^#^ *P* < 0.05 and ^##^ *P* < 0.01 vs. DN model group.

The results showed that the mRNA and protein expression of nephrin, podocin, and WT-1 was significantly downregulated in the DN group compared with the control group (*P* < 0.01) and was significantly upregulated in the two intervention groups compared with the DN group (*P* < 0.01 or 0.05) (Figure 3).

In contrast, the mRNA and protein expression of desmin was significantly upregulated in the DN group compared with the control group (*P* < 0.01) and was significantly downregulated in the two intervention groups compared with the DN group (*P* < 0.01) (Figure 3).

The above results suggest that podocyte injury occurs in the DN model and ACOS can alleviate the podocyte injury.

### 3.6. Effects of ACOS on the Expression of P2X7R/NLRP3 Inflammasome and IL-1*β*/IL-18 in the Renal Cortex of the Rat DN Model

**Figure 4:**
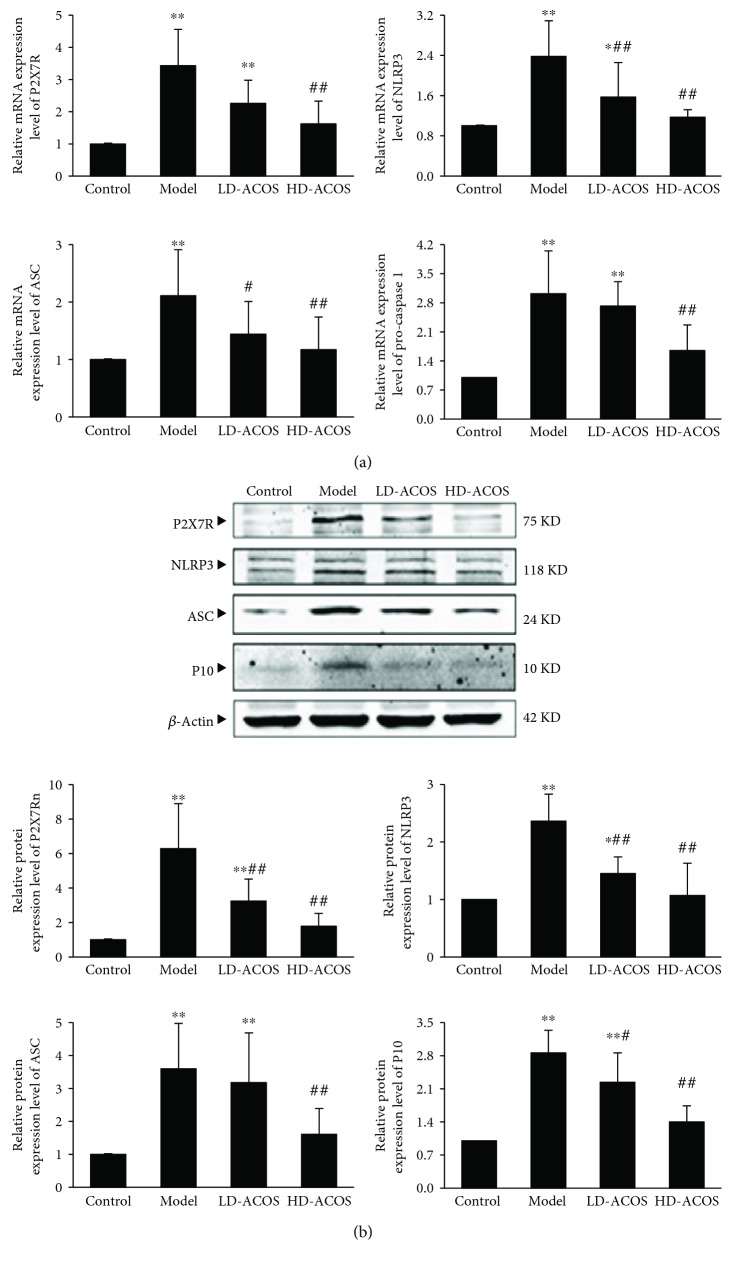
Effects of ACOS on the expression of P2X7R/NLRP3 inflammasome in the rat DN model. (a) Total RNA was extracted from renal cortex tissue, and the relative mRNA expression levels of P2X7R, NLRP3, ASC, and procaspase-1 were measured by real-time quantitative PCR. (b) Renal cortex tissue was lysed, and total lysates were analyzed by the Western blot assay with antibodies against P2X7R, NLRP3, ASC, active caspase-1 subunit P10, and *β*-actin, respectively. The relative protein expression level was expressed as the target protein/*β*-actin ratio. Values are represented as mean ± SD. ^∗^ *P* < 0.05 and ^∗∗^ *P* < 0.01 vs. control group, ^#^ *P* < 0.05 and ^##^ *P* < 0.01 vs. DN model group.

The results showed that the mRNA and protein expression of P2X7R, NLRP3, ASC, and caspase-1 (procaspase-1 mRNA in the gene level and active caspase-1 subunit P10 in the protein level) was significantly upregulated in the DN group compared with the control group (*P* < 0.01), and the above expression was significantly downregulated in the HD-ACOS group compared with the DN group (*P* < 0.01) (Figure 4).

**Figure 5:**
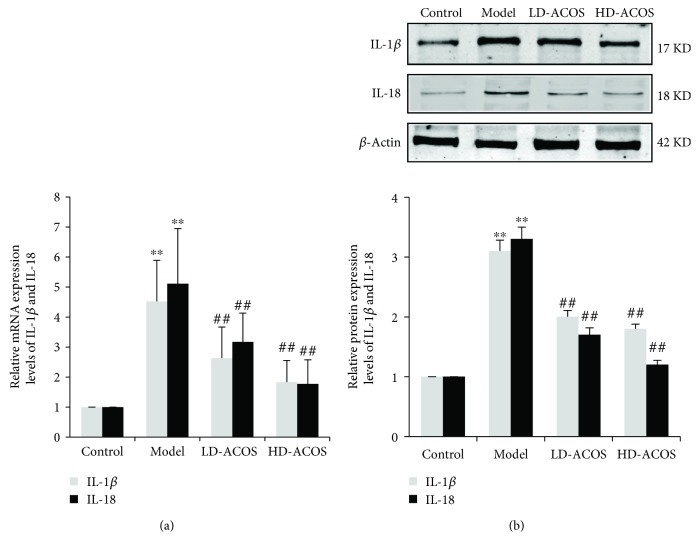
Effects of ACOS on the expression of IL-1*β*/IL-18 in the rat DN model. (a) Total RNA was extracted from renal cortex tissue, and the relative mRNA expression levels of IL-1*β* and IL-18 were measured by real-time quantitative PCR. (b) Renal cortex tissue was lysed, and total lysates were analyzed by the Western blot assay with antibodies against IL-1*β*, IL-18, and *β*-actin, respectively. The relative protein expression level was expressed as the target protein/*β*-actin ratio. Values are represented as mean ± SD. ^∗∗^ *P* < 0.01 vs. control group, ^##^ *P* < 0.01 vs. DN model group.

The mRNA and protein expression of downstream effectors of NLRP3 inflammasome, IL-1*β* and IL-18, was also significantly upregulated in the DN group compared with the control group (*P* < 0.01), and the above expression was significantly downregulated in the HD-ACOS group compared with the DN group (*P* < 0.01) (Figure 5).

The above results suggest that there are enhanced expression and activation of P2X7R/NLRP3 inflammasome and IL-1*β*/IL-18 in the DN model and ACOS can attenuate the changes.

### 3.7. The Localization of P2X7R/NLRP3 in Podocytes of the Rat Kidney in the DN Model

**Figure 6:**
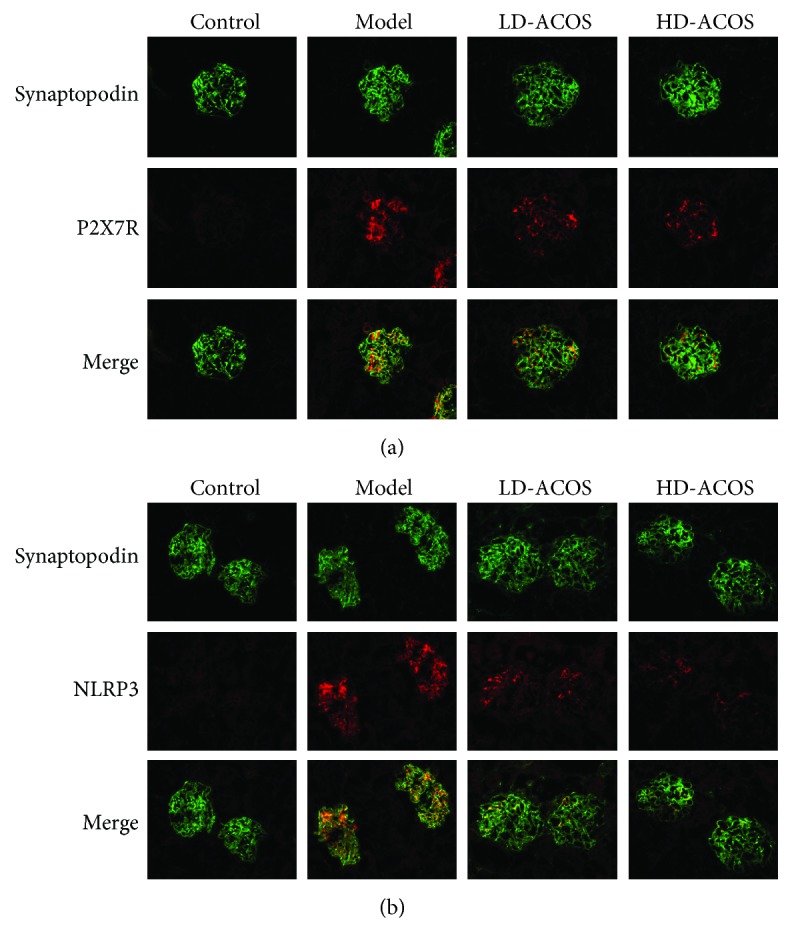
Double immunofluorescence staining of the podocyte marker and P2X7R/NLRP3 in renal tissues of the rat DN model. The colocalization of P2X7R (a, red spots) or NLRP3 (b, red spots) and synaptopodin (a podocyte marker; green spots indicate alone localization and yellow spots indicate colocalization) in the frozen renal tissue section of the rat DN model (immunofluorescence microscopy ×400).

As shown in Figure 6, double immunofluorescence staining analysis revealed that the P2X7R (Figure 6(a), red spots) and NLRP3 (Figure 6(b), red spots) both were colocalized with synaptopodin (a podocyte marker; green spots indicate alone localization and yellow spots indicate colocalization) in glomeruli of DN rats. Outside of glomeruli, there was hardly any expression of P2X7R and NLRP3. Furthermore, the expression levels of P2X7R/NLRP3 in podocytes were obviously decreased in the HD-ACOS and LD-ACOS groups compared with the DN group.

The above results suggest that P2X7R and NLRP3 both are expressed on podocytes and ACOS can weaken their expression.

### 3.8. Effects of ACOS on the HG-Induced Expression of Podocyte-Associated Molecules in Cultured Podocytes

**Figure 7:**
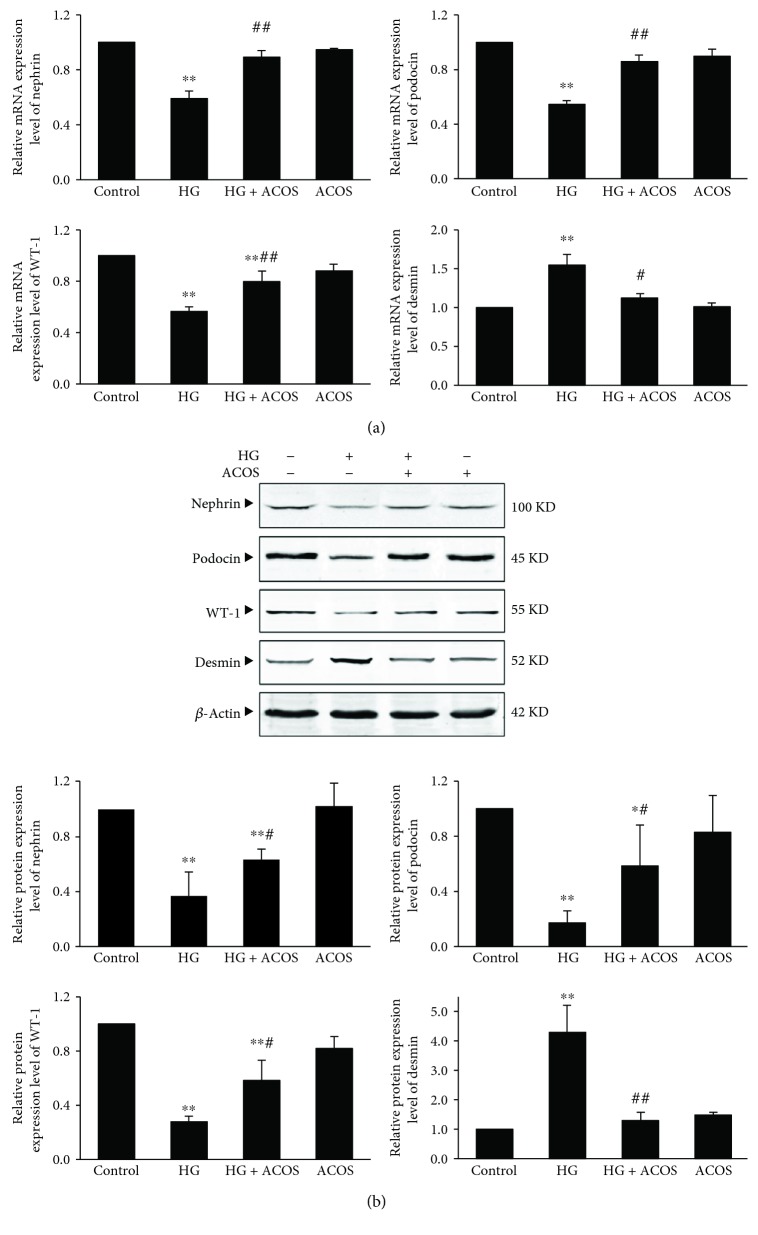
Effects of ACOS on the high-glucose-induced expression of podocyte-associated molecules in cultured podocytes. Podocytes were incubated in medium and medium containing 30 mM glucose and/or 50 *μ*g/mL ACOS, respectively. (a) After 6 h of incubation, cells were harvested. Then, the total RNA was extracted, and the relative mRNA expression levels of nephrin, podocin, WT-1, and desmin of podocytes were measured by real-time quantitative PCR. (b) After 24 h of incubation, cells were lysed and the total lysates were used to determine the protein expression levels of nephrin, podocin, WT-1, desmin, and *β*-actin by the Western blot assay. The relative protein expression level was expressed as the target protein/*β*-actin protein ratio. Values are represented as mean ± SD (*n* = 3). ^∗^ *P* < 0.05 and ^∗∗^ *P* < 0.01 vs. control group, ^#^ *P* < 0.05 and ^##^ *P* < 0.01 vs. HG group.

The results showed that the mRNA and protein expression of nephrin, podocin, and WT-1 was significantly downregulated in the HG group compared with the control group (*P* < 0.01) and was significantly upregulated in the HG + ACOS group compared with the HG group (*P* < 0.01 or 0.05) (Figure 7).

On the contrary, the mRNA and protein expression of desmin was significantly upregulated in the HG group compared with the control group (*P* < 0.01) and was significantly downregulated in the HG + ACOS group compared with the HG group (*P* < 0.01 or 0.05) (Figure 7).

We performed an experiment of parallel controls with mannitol and did not find podocyte injury being caused by HG-related high osmotic pressure (Figure 1S, see Supplementary Materials). The results of the cell experiment are consistent with the results of the animal experiment, and both suggest that ACOS can alleviate the HG-induced podocyte injury.

### 3.9. Effects of ACOS on the HG-Induced Expression of P2X7R/NLRP3 Inflammasome and IL-1*β*/IL-18 in Cultured Podocytes

**Figure 8:**
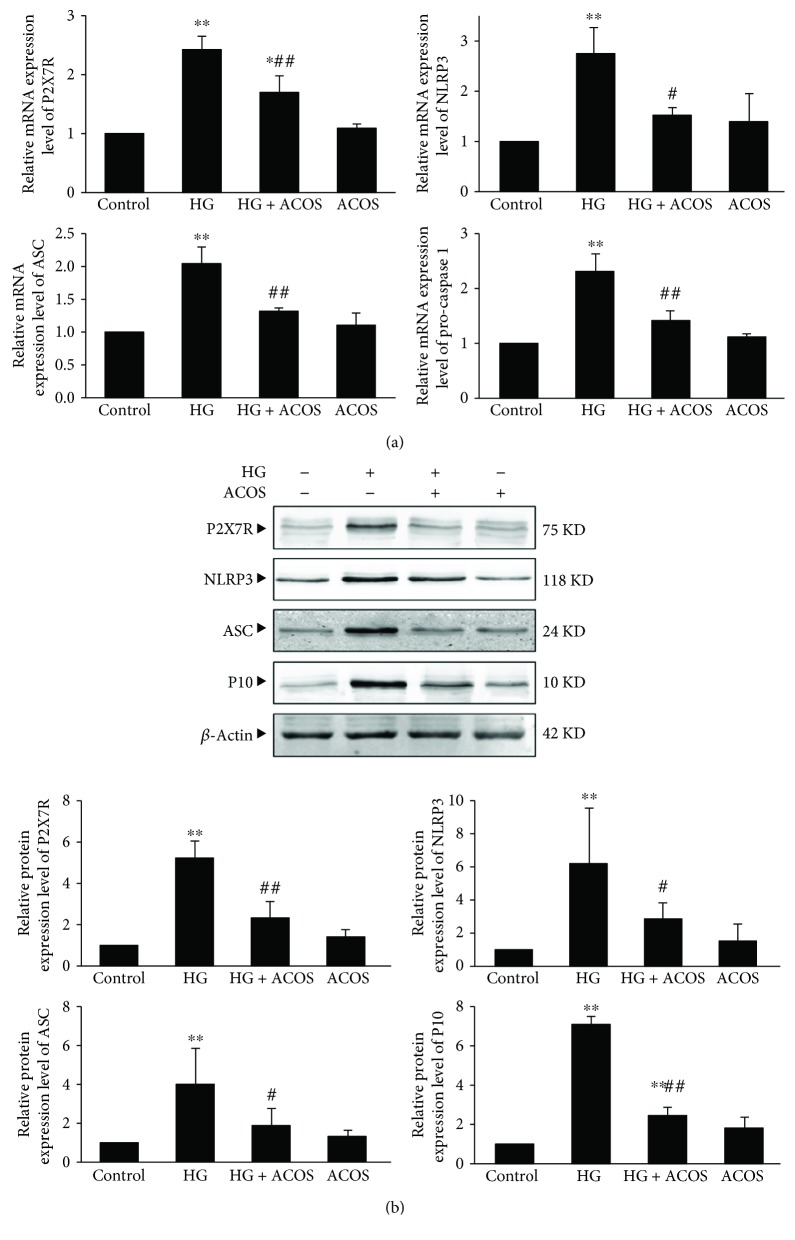
Effects of ACOS on the high-glucose-induced expression of P2X7R/NLRP3 inflammasome in cultured podocytes. Podocytes were incubated in medium and medium containing 30 mM glucose and/or 50 *μ*g/mL ACOS, respectively. (a) After 6 h of incubation, cells were harvested. Then, the total RNA was extracted, and the relative mRNA expression levels of P2X7R, NLRP3, ASC, and procaspase-1 were measured by real-time quantitative PCR. (b) After 24 h of incubation, cells were lysed and the total lysates were used to determine the protein expression levels of P2X7R, NLRP3, ASC, active caspase-1 subunit P10, and *β*-actin by the Western blot assay. The relative protein expression level was expressed as the target protein/*β*-actin protein ratio. Values are represented as mean ± SD (n = 3). ^∗^ *P* < 0.05 and ^∗∗^ *P* < 0.01 vs. control group, ^#^ *P* < 0.05 and ^##^ *P* < 0.01 vs. HG group.

The results showed that the mRNA and protein expression of P2X7R, NLRP3, ASC, and caspase-1 (procaspase-1 mRNA in the gene level and active caspase-1 subunit P10 in the protein level) was significantly upregulated in the HG group compared with the control group (*P* < 0.01), and the above expression was significantly downregulated in the HG + ACOS group compared with the HG group (*P* < 0.01 or 0.05) (Figure 8).

**Figure 9:**
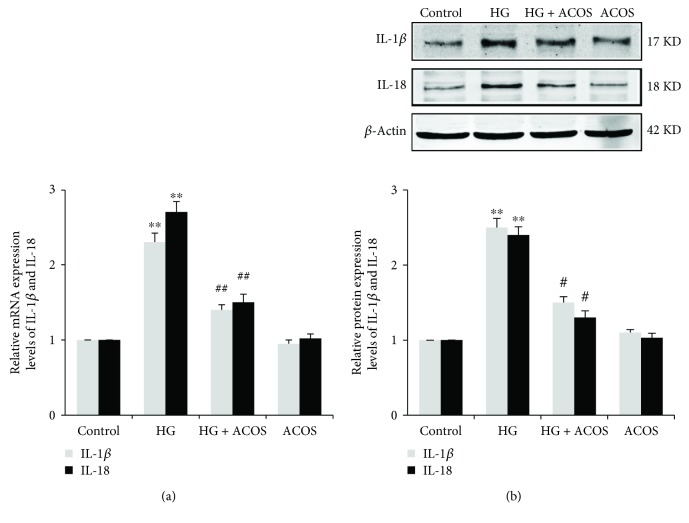
Effects of ACOS on the high-glucose-induced expression of IL-1*β*/IL-18 in cultured podocytes. Podocytes were incubated in medium and medium containing 30 mM glucose and/or 50 *μ*g/mL ACOS, respectively. (a) After 6 h of incubation, cells were harvested. Then, the total RNA was extracted, and the relative mRNA expression levels of IL-1*β* and IL-18 were measured by real-time quantitative PCR. (b) After 24 h of incubation, cells were lysed and the total lysates were used to determine the protein expression levels of IL-1*β*, IL-18, and *β*-actin by the Western blot assay. The relative protein expression level was expressed as the target protein/*β*-actin protein ratio. Values are represented as mean ± SD (*n* = 3). ^∗∗^ *P* < 0.01 vs. control group, ^#^ *P* < 0.05 and ^##^ *P* < 0.01 vs. HG group.

Accordingly, the mRNA and protein expression of IL-1*β* and IL-18 was significantly upregulated in the HG group compared with the control group (*P* < 0.01 or *P* < 0.05), and the above expression was significantly downregulated in the HG + ACOS group compared with the HG group (*P* < 0.01 or 0.05) (Figure 9).

The results of the cell experiment are consistent with the results of the animal experiment, and both suggest that ACOS can attenuate the HG-induced inflammatory reaction in podocytes.

## 4. Discussion

In this study, we successfully established a rat model of DN caused by type 2 DM, which was induced by high-fat-diet feeding and low-dose streptozotocin injection and characterized by insulin resistance and hyperglycemia [25, 26]. In the model, the rats of DN developed obvious proteinuria, elevated serum creatinine, and podocyte injury showing as foot process segmental effacement with an increased width and significant alteration of the expression of podocyte-associated molecules (downregulation of nephrin, podocin, and WT-1 and upregulation of desmin). In addition, we also successfully established a cell model of podocyte injury, which was produced with the stimulation of HG [27]. In the cell model, the expression of podocyte-associated molecules was also significantly changed, which was similar to what is seen in the rat DN model, suggesting podocyte injury. Both the animal and cell models provide good platforms for us to study *in vivo* and *in vitro* the mechanism of podocyte injury of DN and the intervention effects of ACOS.

It has been recognized that podocyte injury is a pivotal event in the pathogenesis of DN, which contributes to the occurrence and progression of DN [4]. The podocyte injury in DN includes decreased podocyte density and number, foot process effacement, detachment and disruption of the slit diaphragm, podocyte loss and its resulting hypertrophy and/or dedifferentiation, and podocyte death [4]. The above changes of podocytes will cause proteinuria, while the proteinuria itself can further aggravate the podocyte injury to form a vicious cycle, eventually leading to glomerulosclerosis and renal dysfunction [4]. Therefore, in this study, we focused on watching the changes of podocytes in the rat DN model and in the HG-stressed cell model.

In the past years, the pathogenic role of P2X7R and NLRP3 inflammasome in kidney diseases was highly noticed [28, 29], and the expression of P2X7R and all the components of NLRP3 inflammasome in podocytes was also affirmed [8, 30, 31]. In 2004, Vonend et al. [8] first reported that the expression of P2X7R in glomeruli, mainly in podocytes, was significantly upregulated in a diabetic rat model. In 2014, Shahzad et al. [7] observed that the NLRP3 inflammasome was activated in the podocytes of the patients with DN and the mouse models of DN and in the glucose-stressed podocytes in culture. Our studies *in vivo* and *in vitro* further confirm their previous findings. Furthermore, because P2X7R is a potent activator of the NLRP3 inflammasome [32], we put them together to watch their changes in the studies *in vivo* and *in vitro* and found that the upregulation of P2X7R expression was always synchronized with the activation of NLRP3 inflammasome and its downstream effectors IL-1*β* and IL-18. So, our study extends, to a certain extent, the previous observations.

*Ophiocordyceps sinensis* and its anamorph *Hirsutella sinensis* are widely used in the treatment of kidney diseases in China and have been proven to be effective in the treatment of DN both in clinical practice and in animal experiments [16]. In this study, our experiments with ACOS also affirmed its effectiveness. Moreover, through the experiments *in vivo* and *in vitro*, we found that *Ophiocordyceps sinensis* could significantly inhibit the high expression of P2X7R and the activation of NLRP3 inflammasome in podocytes, which may be one of the important mechanisms for the therapeutic effects of *Ophiocordyceps sinensis* on DN. To our knowledge, no similar research results have been published so far. Our study also showed that ACOS could improve insulin resistance and reduce blood glucose level in a diabetic rat model, which is consistent with previous studies [16, 18, 33]. Undoubtedly, the effects can also contribute to the prevention and treatment of DN.

Wild *Ophiocordyceps sinensis* only grows in China's Qinghai-Tibet Plateau, and its resource is quite limited. The predatory digging and collecting every year not only exhaust the resources but also destroy the fragile ecological environment on the local plateau. Now, wild *Ophiocordyceps sinensis* has been listed by the Chinese government as “National Grade II Protected Species” and has been restricted from picking [19, 20]. So, artificial cultivation of *Ophiocordyceps sinensis* is urgently needed. After more than 30 years of hard work to explore and overcome a lot of technical difficulties, artificial cultivation and industrialized production of *Ophiocordyceps sinensis* were finally successful in 2015 [20], and the ACOS has been verified by the Chinese Academy of Sciences [21]. After molecular systematic analysis [21], nuclear magnetic resonance fingerprint analysis [34], and chemical compound analysis [35, 36], it has been confirmed that the ACOS has no significant difference with wild *Ophiocordyceps sinensis* and its quality fully complies with the standard of Chinese pharmacopoeia. Our study is the first experimental research to treat the disease with ACOS and has gained good results, which clearly displays that the ACOS is effective for the treatment of DN.

In conclusion, our experiments *in vivo* and *in vitro* conducted in the DN rat model and HG-stressed podocyte model suggest that P2X7R and NLRP3 inflammasome are involved in the pathogenesis of DN including its podocyte injury and *Ophiocordyceps sinensis* including ACOS can effectively alleviate the podocyte injury of DN. The therapeutic efficacy may be related to its antagonistic effects on the P2X7R/NLRP3 inflammasome axis. The above research results provide a valuable experimental basis for the clinical application of ACOS in the prevention and treatment of DN.

## Figures and Tables

**Table 1:** Primer sequences for PCR analysis in animal experiments.

Target	Primer sequence (5′–3′)	Length (bp)
Nephrin	Forward CTGGGGGACAGTGGATTGAC	158
	Reverse CCACCAACTGCAAAGAGCAC	
Podocin	Forward GCTGTCTGCTACTACCGCAT	125
	Reverse GTGAGGGATCGATGTGCCAA	
WT-1	Forward TGCGTCCTATCAGGTTTGCC	188
	Reverse CCCAGCGAAATGAGCACAAG	
Desmin	Forward CTGAGCAAAGGGGTTCCGA	185
	Reverse GTGTGACATCCGAGAGTGGA	
P2X7R	Forward TAACCACTGAGCCGTCCCTA	127
	Reverse AGGTCCTCATGCTTGTGCTC	
NLRP3	Forward TAGCTTCTGCCGAGGTCTCT	176
	Reverse ATTGATGGGTCAGTCCGCAG	
ASC	Forward ACAGTACCAGGCAGTTCGTG	143
	Reverse GGTCTGTCACCAAGTAGGGC	
Procaspase-1	Forward CCGGGGATCCCTCTTCATTG	144
	Reverse ACCCTTTCAGTGGTTGGCAT	
IL-1*β*	Forward AGGCTGACAGACCCCAAAAG	178
	Reverse CTCCACGGGCAAGACATAGG	
IL-18	Forward ACCGCAGTAATACGGAGCAT	110
	Reverse GTCTGGGATTCGTTGGCTGT	
GAPDH	Forward TGGGTGTGAACCACGAGAA	143
	Reverse GGCATGGACTGTGGTCATGA	

**Table 2:** Primer sequences for PCR analysis in cellular experiments.

Target	Primer sequence (5′–3′)	Length (bp)
Nephrin	Forward GTCTGGGGACCCCTCTATGA	209
	Reverse CAGGTCTTCTCCAAGGCTGT	
Podocin	Forward CAGAAGGGGAAAAGGCTGCT	200
	Reverse GATGCTCCCTTGTGCTCTGT	
WT-1	Forward TCCGGTCAGCATCTGAAACC	179
	Reverse GAGCTGGTCTGAGCGAGAAA	
Desmin	Forward GTTTCAGACTTGACTCAGGCAG	106
	Reverse TCTCGCAGGTGTAGGACTGG	
P2X7R	Forward CACCGTGCTTACAGGTGCTA	115
	Reverse CGGTCTTGGGGAACTCCTTC	
NLRP3	Forward TCTGCACCCGGACTGTAAAC	131
	Reverse CATTGTTGCCCAGGTTCAGC	
ASC	Forward GACAGTGCAACTGCGAGAAG	106
	Reverse CGACTCCAGATAGTAGCTGACAA	
Procaspase-1	Forward ACAAGGCACGGGACCTATG	237
	Reverse TCCCAGTCAGTCCTGGAAATG	
IL-1*β*	Forward CGCAGCAGCACATCAACAAG	118
	Reverse GTGCTCATGTCCTCATCCTG	
IL-18	Forward ACTTTGGCCGACTTCACTGT	135
	Reverse GTCTGGTCTGGGGTTCACTG	
GAPDH	Forward TGTGAACGGATTTGGCCGTA	202
	Reverse GATGGGCTTCCCGTTGATGA	

**Table 3:** Primary and secondary antibodies for Western blot assays.

Primary antibody	Secondary antibody
Rabbit anti-nephrin pAb (Abcam)	IRDye 800-conjugated goat anti-rabbit IgG antibody (LI-COR)
Rabbit anti-podocin pAb (Sigma)	Ditto
Rabbit anti-WT-1 pAb (Abcam)	Ditto
Rabbit anti-desmin pAb (Abcam)	Ditto
Rabbit anti-P2X7R pAb (Alomone)	Ditto
Rabbit anti-NLRP3 pAb (Novus)	Ditto
Rabbit anti-ASC pAb (Santa Cruz)	Ditto
Rabbit anti-caspase-P10 pAb (Santa Cruz)	Ditto
Rabbit anti-IL-1*β* pAb (Abcam)	Ditto
Mouse anti-IL-18 mAb (Santa Cruz)	IRDye 680-conjugated goat anti-mouse IgG antibody (LI-COR)
Mouse anti-*β*-actin mAb (Sigma)	Ditto

**Table 4:** Biological parameters in the different groups at the 13th week ($\bar{x}\pm s$).

Group	BW (g)	KW/BW (mg/g)	FBG (mmol/L)	HbA1c (%)	SCr (*μ*mol/L)	Upro (mg/d)
Control	526.9 ± 51.5	6.1 ± 0.3	6.95 ± 0.53	11.8 ± 0.7	24.9 ± 2.6	6.0 ± 1.5
Model	452.9 ± 41.1^∗^	8.8 ± 1.5^∗^	32.58 ± 1.31^∗∗^	20.3 ± 0.9^∗∗^	34.8 ± 4.9^∗^	33.8 ± 9.7^∗^
LD-ACOS	438.7 ± 50.1^∗^	8.5 ± 1.1^∗^	30.96 ± 4.03^∗∗^	20.0 ± 1.1^∗∗^	32.8 ± 4.2^∗^	24.6 ± 8.1^∗^^#^
HD-ACOS	466.1 ± 105.3	7.4 ± 1.1^∗^^#^	20.25 ± 7.86^∗∗^^##^	18.4 ± 3.2^∗∗^	28.9 ± 2.5^∗^^#^	18.4 ± 4.6^∗^^#^

^∗^ *P* < 0.05 and ^∗∗^ *P* < 0.01 vs. control group; ^#^ *P* < 0.05 and ^##^ *P* < 0.01 vs. model group. BW: body weight; KW: kidney weight; FBG: fasting blood glucose; HbA1c: glycosylated hemoglobin A1c; SCr: serum creatinine; Upro: urinary protein.

**Table 5:** Serum insulin and insulin resistance index in different groups ($\bar{x}\pm s$).

Group	4th week		13th week	
	Serum insulin (mIU/L)	IRI	Serum insulin (mIU/L)	IRI
Control	18.6 ± 1.5	5.03 ± 0.56	20.7 ± 2.2	6.43 ± 1.99
Model	23.9 ± 2.6^∗^	6.52 ± 1.29^∗^	17.2 ± 1.4^∗^	24.89 ± 2.81^∗∗^
LD-ACOS	24.5 ± 4.2^∗^	6.49 ± 0.85^∗^	19.4 ± 2.2	24.47 ± 2.55^∗∗^
HD-ACOS	24.3 ± 3.0^∗^	6.48 ± 2.11^∗^	20.6 ± 2.3^#^	18.49 ± 7.33^∗∗^^##^

^∗^ *P* < 0.05 and ^∗∗^ *P* < 0.01 vs. control group; ^#^ *P* < 0.05 and ^##^ *P* < 0.01 vs. model group. IRI: insulin resistance index.

## Data Availability

The original data of the current study are available in the following website: https://pan.baidu.com/s/16Np4-nJjQjtovUhCdYdzcQ.

## Supplementary Materials

Supplementary Materials Table 1S: the cytotoxicity of high glucose (HG) on cultured podocytes. Figure 1S: effects of mannitol or high glucose on the expression of podocyte-associated molecules in cultured podocytes.
