# Supplementary material for: Artificially Cultivated Ophiocordyceps sinensis Alleviates Diabetic Nephropathy and Its Podocyte Injury via Inhibiting P2X7R Expression and NLRP3 Inflammasome Activation
Source: J Diabetes Res. 2018 Nov 11;2018:1390418. doi: 10.1155/2018/1390418 (PMC6252193; doi:10.1155/2018/1390418)
Supplement: Supplementary Materials — Table 1S: the cytotoxicity of high glucose (HG) on cultured podocytes. Figure 1S: effects of mannitol or high glucose on the expression of podocyte-associated molecules in cultured podocytes. [file 1390418.f1.pdf]

Supplementary data

The cytotoxicity test of high glucose (HG) on cultured podocytes

To evaluate the cytotoxicity of HG on cultured podocytes, the release test of lactate dehydrogenase (LDH), a cytoplasmic enzyme, was performed. The activity of LDH in the cell culture supernatant was measured with a CytoTox 96® Non-Radioactive Cytotoxicity Assay Kit (Promega, USA) and the LDH release rate (%) was calculated according to the instruction manual of the kit.

Cultured podocytes were treated with glucose of different concentrations (0, 5, 15, 30 and 60 mM) for 36h. Results showed that either 5mM, 15mM, 30mM or 60 mM glucose had no cytotoxic effects on cellular viability (Table 1S). According to the above results of LDH release test, 30mM glucose was chosen as the concentration of HG stimulating podocytes in all experiments.

Table 1S. The cytotoxicity of high glucose (HG) on cultured podocytes

Concertration of glucose	*0mM*	*5mM*	*15mM*	*30mM*	*60mM*
LDH release rate (%)	7.6±1.1	8.7±1.3	7.7±1.5	7.3±1.7	8.8±1.2

Effects of mannitol or high glucose on expression of podocyte- associated molecules in cultured podocytes

Cultured podocytes were incubated in medium, medium containing 25mM mannitol or medium containing 30mM glucose, respectively. 25mM mannitol was served as a control of high osmolarity. After 6 h of incubation, cells were harvested. Then, the total RNA was extracted and the relative mRNA expression levels of nephrin, podocin, WT-1 and desmin were measured by real time quantitative PCR. B: After 24 h of incubation, cells were lysed and the total lysates were used to determine the protein expression levels of nephrin, podocin, WT-1, desmin and β-actin by Western blot assay. The relative protein expression level was expressed as the target protein/β-actin protein ratio.

The results of the above experimentals are showed in Figure 1S. A： the results of real time quantitative PCR; B: the results of Western blot assay. Values are represented as mean ± SD (n=3). **P＜0.01 vs. control group.

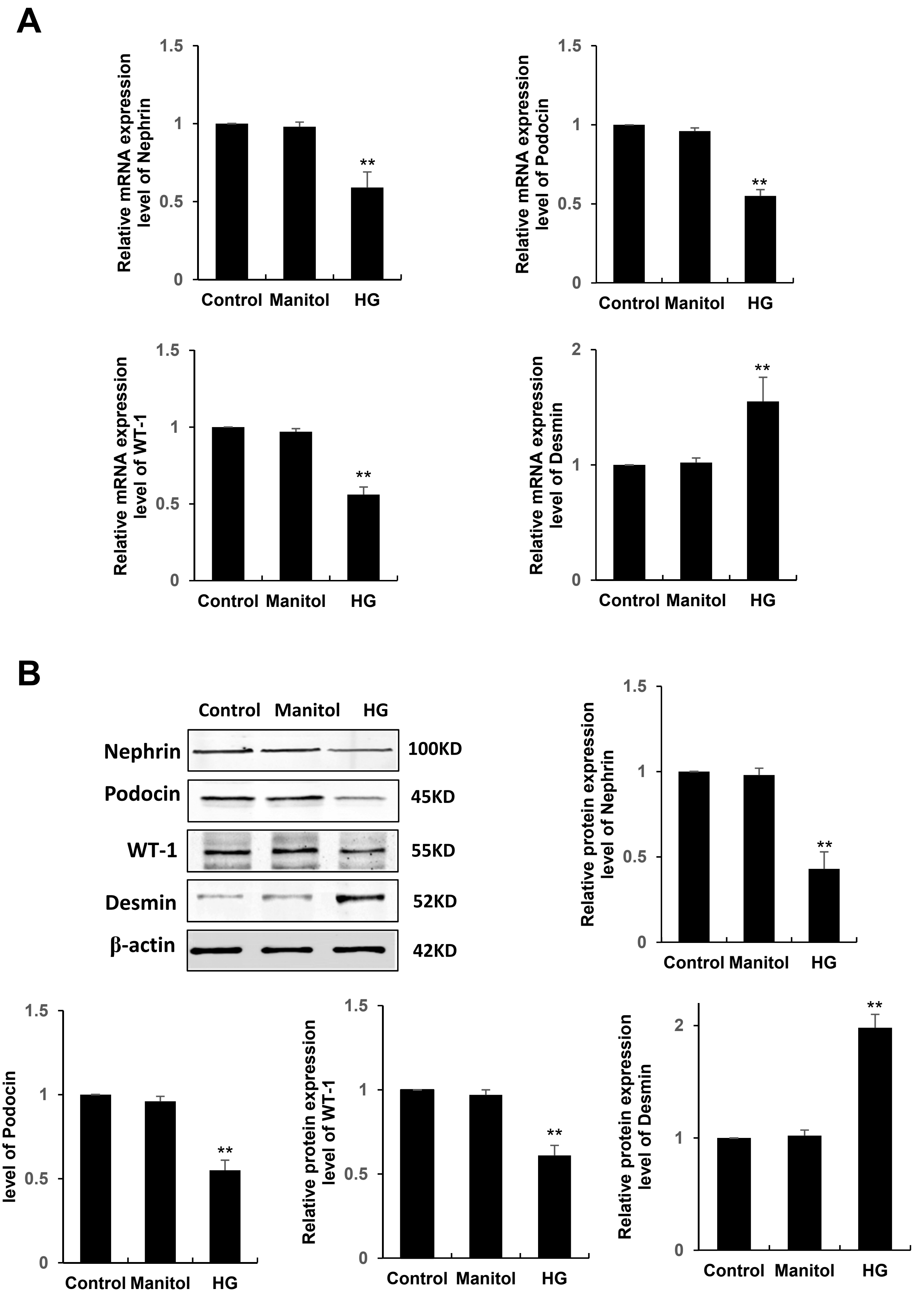
A
Relative mRNA expression level of Nephrin
Relative mRNA expression level of Podocin
Relative mRNA expression level of WT-1
Relative mRNA expression level of Desmin
Control Manitol HG
**
B
Control Manitol HG
Nephrin 100KD
Podocin 45KD
WT-1 55KD
Desmin 52KD
β-actin 42KD
Relative protein expression level of Nephrin
Relative protein expression level of Podocin
Relative protein expression level of WT-1
Relative protein expression level of Desmin

Figure 1S
